# A Perspective on Explanations of Molecular Prediction Models

**DOI:** 10.1021/acs.jctc.2c01235

**Published:** 2023-03-27

**Authors:** Geemi P. Wellawatte, Heta A. Gandhi, Aditi Seshadri, Andrew D. White

**Affiliations:** † Department of Chemistry, University of Rochester, Rochester, New York 14627, United States; ‡ Department of Chemical Engineering, University of Rochester, Rochester, New York 14627, United States

## Abstract

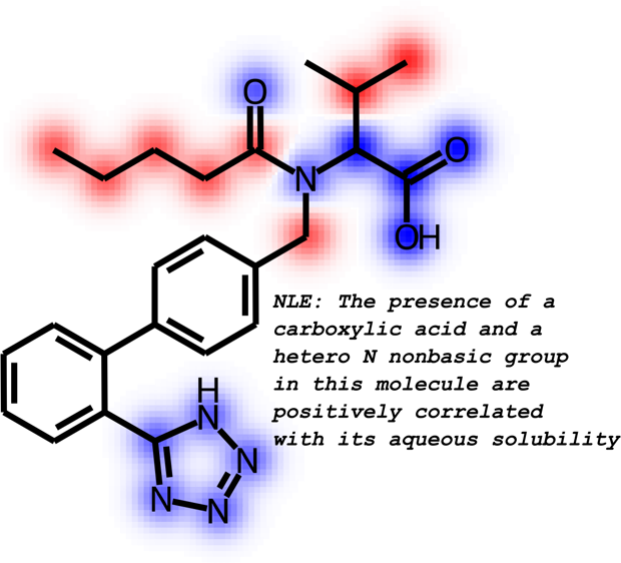

Chemists can be skeptical in using deep learning (DL) in decision making, due to the lack of interpretability in “black-box” models. Explainable artificial intelligence (XAI) is a branch of artificial intelligence (AI) which addresses this drawback by providing tools to interpret DL models and their predictions. We review the principles of XAI in the domain of chemistry and emerging methods for creating and evaluating explanations. Then, we focus on methods developed by our group and their applications in predicting solubility, blood–brain barrier permeability, and the scent of molecules. We show that XAI methods like chemical counterfactuals and descriptor explanations can explain DL predictions while giving insight into structure–property relationships. Finally, we discuss how a two-step process of developing a black-box model and explaining predictions can uncover structure–property relationships.

## Introduction

Deep learning (DL) is advancing the boundaries of computational chemistry because it can accurately model nonlinear structure–function relationships.^1−3^ Applications of DL can be found in a broad spectrum spanning from quantum computing^4,5^ to drug discovery^6−10^ to materials design.^11,12^ DL models can contribute to scientific discovery in three “dimensions”: (1) as a “computational microscope” to gain insight which is not attainable through experiments, (2) as a “resource of inspiration” to motivate scientific thinking; (3) as an “agent of understanding” to uncover new observations.^13^ However, the rationale of a DL prediction is not always apparent due to the model architecture consisting of a large parameter count.^14,15^ DL models are thus often termed “black-box” models. We can only reason about the input and output of a DL model, not the underlying cause that leads to a specific prediction.

It is routine in chemistry now for DL to exceed human level performance (humans are not good at predicting solubility from structure, for example;^16^ there does happen to be one human solubility savant, participant 11, who matched machine performance), and so, understanding how a model makes predictions can guide hypotheses. This is in contrast to a topic like finding a stop sign in an image, where there is little new to be learned about visual perception by explaining a DL model. However, the black-box nature of DL has its own limitations. Users are more likely to trust and use predictions from a model if they can understand why the prediction was made.^17^ Explaining predictions can help developers of DL models ensure the model is not learning spurious correlations.^18,19^ Two infamous examples are (1) neural networks that learned to recognize horses by looking for a photographer’s watermark^20^ and (2) neural networks that predicted a COVID-19 diagnosis by looking at the font choice on medical images.^21^ As a result, there is an emerging regulatory framework for when any computer algorithms impact humans.^22−24^ Although we know of no examples yet in chemistry, one can assume the use of artificial intelligence (AI) in predicting toxicity, carcinogenicity, and environmental persistence will require rationale for the predictions due to regulatory consequences.

Explainable artificial intelligence (XAI) is a field of growing importance that aims to provide model interpretations of DL predictions. Three terms highly associated with XAI are interpretability, justifications, and explainability. Miller^25^ defines that interpretability of a model refers to the degree of human understandability intrinsic within the model. Murdoch, Singh, Kumbier, Abbasi-Asl, and Yu^26^ clarify that interpretability can be perceived as “knowledge”, which provides insight into a particular problem. Justifications are quantitative metrics that tell the users “why the model should be trusted”, like test error.^27^ Justifications are evidence which defend why a prediction is trustworthy.^25^ An “explanation” is a description of why a certain prediction was made.^9,28^ Interpretability and explanation are often used interchangeably. Furthermore, it is distinguished that interpretability is a passive characteristic of a model, whereas explainability is an active characteristic, which is used to clarify the internal decision-making process.^14^ In other words, an explanation is extra information that gives the context and a cause for one or more predictions.^29^ We adopt the same nomenclature in this Review.

Accuracy and interpretability are two attractive characteristics of DL models. However, DL models are often highly accurate and less interpretable.^28,30^ XAI provides a way to avoid that trade-off in chemical property prediction. XAI can be viewed as a two-step process. First, we develop an accurate but uninterpretable DL model. Next, we add explanations to predictions. Ideally, if the DL model has correctly learned the input–output relations, then the explanations should give insight into the underlying mechanism.

In the remainder of this Review, we review recent approaches for XAI of chemical property prediction while drawing specific examples from our recent XAI work.^9,10,31^ We show how in various systems these methods yield explanations that are consistent with known mechanisms in structure–property relationships.

## Theory

In this work, we aim to assemble a common taxonomy for the landscape of XAI while providing our perspectives. We utilized the vocabulary proposed by Das and Rad^32^ to classify XAI. According to their classification, interpretations can be categorized as global or local interpretations on the basis of “what is being explained?”. For example, counterfactuals are local interpretations, as these can explain only a given instance. The second classification is based on the relation between the model and the interpretation: is interpretability posthoc (extrinsic) or intrinsic to the model?^32,33^ An intrinsic XAI method is part of the model and is self-explanatory.^32^ These are also referred to as white-box models to contrast them with noninterpretable black-box models.^28^ An extrinsic method is one that can be applied post-training to any model.^33^ Posthoc methods found in the literature focus on interpreting models through (1) training data^34^ and feature attribution,^35^ (2) surrogate models^10^ and, (3) counterfactual^9^ or contrastive explanations.^36^

Often, what is a “good” explanation and what are the required components of an explanation are debated.^32,37,38^ Palacio, Lucieri, Munir, Ahmed, Hees, and Dengel^29^ state that the lack of a standard framework has caused the inability to evaluate the interpretability of a model. In the physical sciences, we may instead consider if the explanations somehow reflect and expand our understanding of physical phenomena. For example, Oviedo, Ferres, Buonassisi, and Butler^39^ propose that a model explanation can be evaluated by considering its agreement with physical observations, which they term “correctness”. For example, if an explanation suggests that polarity affects the solubility of a molecule and the experimental evidence strengthens the hypothesis, then the explanation is assumed to be “correct”. In instances where such mechanistic knowledge is sparse, expert biases and subjectivity can be used to measure the correctness.^40^ Other similar metrics of correctness such as “explanation satisfaction scale” can be found in the literature.^41,42^

Based on the above discussion, we identify that an agreed upon evaluation metric is necessary in XAI. We suggest the following attributes can be used to evaluate explanations. However, the relative importance of each attribute may depend on the application: actionability may not be as important as faithfulness when evaluating the interpretability of a static physics-based model. Therefore, one can select the relative importance of each attribute based on the application.

- *Actionable*: Is it clear how we could change the input features to modify the output?
- *Complete*: Does the explanation completely account for the prediction? Did features not included in the explanation really contribute zero effect to the prediction?^43^
- *Correct*: Does the explanation agree with the hypothesized or known underlying physical mechanism?^39^
- *Domain Applicable*: Does the explanation use language and concepts of domain experts?
- *Fidelity/Faithful:* Does the explanation agree with the black-box model?
- *Robust:* Does the explanation change significantly with small changes to the model or the instance being explained?
- *Sparse/Succinct:* Is the explanation succinct?

We present an example evaluation of the SHAP explanation method based on the above attributes.^43^ Shapley values were proposed as a local explanation method based on feature attribution, as they offer a complete explanation: each feature is assigned a fraction of the prediction value.^43,44^ Completeness is a clearly measurable and well-defined metric but yields explanations with many components. Yet, Shapley values are neither actionable nor sparse. They are nonsparse as every feature has a nonzero attribution and not actionable because they do not provide a set of features which change the outcome.^45^ Ribeiro, Singh, and Guestrin^35^ proposed a surrogate model method that aims to provide sparse/succinct explanations that have high fidelity to the original model. In ref (9), we argue that counterfactuals are “better” explanations because they are actionable and sparse. We highlight that the evaluation of explanations is a difficult task because explanations are fundamentally for and by humans. Therefore, these evaluations are subjective, as they depend on “complex human factors and application scenarios”.^37^

### Self-Explaining Models

A self-explanatory model is one that is intrinsically interpretable to an expert.^46^ Two common examples found in the literature are linear regression models and decision trees (DTs). Intrinsic models can be found in other XAI applications acting as surrogate models (proxy models) due to their transparent nature.^47,48^ A linear model is described by eq 1 where, *W* is the weight parameter and *x* is the input feature associated with the prediction *ŷ*. Therefore, the weights can be used to derive a complete explanation of the model: trained weights quantify the importance of each feature.^46^ DT models are another type of self-explaining models which have been used in classification and high-throughput screening tasks. DT models have been used to classify nanomaterials that identify structural features responsible for surface activity.^49^ In another study by Han, Wang, and Bryant,^50^ a DT model was developed to filter compounds by their bioactivity based on chemical fingerprints.
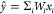
1

Regularization techniques such as EXPO (Explanation-based Optimization)^51^ and RRR (Right for the Right Reasons)^52,53^ are designed to enhance the black-box model interpretability.^54^ Although one can argue that “simplicity” of models is positively correlated with interpretability, this is based on how the interpretability is evaluated. For example, Lipton^55^ argues that, from the notion of “simulatability” (the degree to which a human can predict the outcome based on inputs), self-explanatory linear models, rule-based systems, and DTs can be claimed uninterpretable. A human can predict the outcome given the inputs only if the input features are interpretable. Therefore, a linear model which takes in nondescriptive inputs may not be as transparent. On the other hand, linear models are not innately accurate as they fail to capture nonlinear relationships in data, limiting their applicability. Similarly, a DT is a rule-based model and lacks physics informed knowledge. Therefore, an existing drawback is the trade-off between the degree of understandability and the accuracy of a model. For example, an intrinsic model (linear regression or decision trees) can be described through the trainable parameters, but it may fail to “correctly” capture nonlinear relations in the data.

### Attribution Methods

Feature attribution methods explain black-box predictions by assigning each input feature a numerical value, which indicates its importance or contribution to the prediction. Feature attributions provide local explanations but can be averaged or combined to explain multiple instances. Atom-based numerical assignments are commonly referred to as heatmaps.^56^ Sheridan^57^ describes an atom-wise attribution method for interpreting QSAR models. Recently, Rasmussen, Christensen, and Jensen^58^ showed that Crippen logP models serve as a benchmark for heatmap approaches. Other most widely used feature attribution approaches in the literature are gradient-based methods,^59,60^ Shapley Additive exPlanations (SHAP),^43^ and layerwise relevance prorogation.^61^

Gradient-based approaches are based on the hypothesis that gradients for neural networks are analogous to coefficients for regression models.^62^ Class activation maps (CAMs),^63^ gradCAM,^64^ smoothGrad,^59^ and integrated gradients^62^ are examples of this method. The main idea behind feature attributions with gradients can be represented with eq 2.
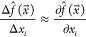
2where *f̂*(*x*) is the black-box model and  is used as our attribution. The left-hand side of eq 2 says that we attribute each input feature *x*_*i*_ by how much one unit change in it would affect the output of *f̂*(*x*). If *f̂*(*x*) is a linear surrogate model, then this method reconciles with LIME.^35^ In DL models, ∇_*x*_*f*(*x*) suffers from the shattered gradient problem.^62^ This means directly computing the quantity leads to numeric problems. The different gradient-based approaches are mostly distinguishable based on how the gradient is approximated.

Gradient-based explanations have been widely used to interpret chemistry predictions.^60,65−69^ McCloskey, Taly, Monti, Brenner, and Colwell^60^ used graph convolutional networks (GCNs) to predict protein–ligand binding and explained the binding logic for these predictions using integrated gradients. References (65 and 66) show applications of gradCAM and integrated gradients to explain molecular property predictions from trained graph neural networks (GNNs). Reference (67) presents comprehensive, open-source XAI benchmarks to explain GNNs and other graph-based models. The authors compared the performance of class activation maps (CAM),^63^ gradCAM,^64^ smoothGrad,^59^ integrated gradients,^62^ and attention mechanisms for explaining outcomes of classification as well as regression tasks. They concluded that CAM and integrated gradients perform well for graph-based models. Another attempt at creating XAI benchmarks for graph models was made by Rao, Zheng, Lu, and Yang.^69^ They compared these gradient-based methods to find subgraph importance when predicting activity cliffs and concluded that gradCAM and integrated gradients provided the most interpretability for GNNs. The GNNExplainer^68^ is an approach for generating explanations (local and global) for graph-based models. This method focus on identifying which subgraphs contribute most to the prediction by maximizing mutual information between the prediction and distribution of all possible subgraphs. It was shown that GNNExplainer can be used to obtain model-agnostic explanations.^68^ SubgraphX is a similar method that explains GNN predictions by identifying important subgraphs.^70^

Another set of approaches like DeepLIFT^71^ and Layerwise Relevance backPropagation^72^ (LRP) are based on backpropagation of the prediction scores through each layer of the neural network. The specific backpropagation logic across various activation functions differs in these approaches, which means each layer must have its own implementation. Baldassarre and Azizpour^73^ showed application of LRP to explain aqueous solubility prediction for molecules.

SHAP is a model-agnostic feature attribution method that is inspired from the game theory concept of Shapley values.^43,45^ SHAP has been popularly used in explaining molecular prediction models.^74−77^ It is an additive feature contribution approach, which assumes that an explanation model is a linear combination of binary variables *z*. If the Shapley value for the *i*^th^ feature is ϕ_*i*_, then the explanation is *f̂*(*x⃗*) = Σ_*i*_ϕ_*i*_(*x⃗*)*z*_*i*_(*x⃗*). Shapley values for features are computed using eq 3.^78,79^
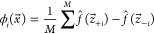
3Here, *z⃗* is a fabricated example created from the original *x⃗* and a random perturbation *x⃗*′. *z⃗*_+*i*_ has the feature *i* from *x⃗*, and *z⃗*–*i* has the *i*^th^ feature from *x⃗*′. Some care should be taken in constructing *z⃗* when working with molecular descriptors to ensure that an impossible *z⃗* is not sampled (e.g., high count of acid groups but no hydrogen bond donors). *M* is the sample size of perturbations around *x⃗*. Shapley value computation is expensive; hence, *M* is chosen accordingly. Equation 3 is an approximation and gives contributions with an expectation term as ϕ_0_ + Σ_*i*=1_ ϕ_*i*_(*x⃗*) = *f̂*(*x⃗*).

Visualization-based feature attribution has also been used for molecular data. In computer science, saliency maps are a way to measure the spatial feature contribution.^80^ Simply put, saliency maps draw a connection between the model’s neural fingerprint components (trained weights) and input features. In ref (81), saliency maps were used to build an explainable GCN architecture that gives subgraph importance for small molecule activity prediction. On the other hand, similarity maps compare model predictions for two or more molecules based on their chemical fingerprints.^82^ Similarity maps provide atomic weights or predicted probability differences between the molecules by removing one atom at a time. These weights can then be used to color the molecular graph and give a visual presentation. ChemInformatics Model Explorer (CIME) is an interactive web-based toolkit which allows visualization and comparison of different explanation methods for molecular property prediction models.^83^

### Surrogate Models

One approach to explain black-box predictions is to fit a self-explaining or interpretable model to the black-box model in the vicinity of one or a few specific examples. These are known as surrogate models. Generally, one model per explanation is trained. However, if we could find one surrogate model that explained the whole DL model, then we would simply have a globally accurate interpretable model. This means that the black-box model is no longer needed.^78^ In work by Gandhi and White,^10^ a weighted least-squares linear model is used as the surrogate model. Our approach provides natural language-based descriptor explanations by replacing input features with chemically interpretable descriptors. This approach is similar to the concept-based explanations approach used in ref (84), where human understandable concepts are used in place of input features in acquisition of chess knowledge in AlphaZero. Any of the self-explaining models detailed in the Self-Explaining Models section can be used as a surrogate model.

The most commonly used surrogate model-based method is Locally Interpretable Model Explanations (LIME).^35^ LIME created perturbations around the example of interest and fits an interpretable model to these local perturbations. An explanation ξ is mathematically defined for an example *x⃗* using eq 4.^35^
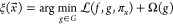
4Here, *f* is the black-box model and *g*∈*G* is the interpretable explanation model. *G* is a class of potential interpretable models (e.g., linear models). π_*x*_ is a similarity measure between original input *x⃗* and its perturbed input *x⃗*′. In the context of molecular data, this can be a chemical similarity metric like Tanimoto^85^ similarity between fingerprints. The goal for LIME is to minimize the loss, , such that *f* is closely approximated by *g*. Ω is a parameter that controls the complexity (sparsity) of *g*. The agreement (how low the loss is) between *f* and *g* is termed “fidelity”.^35^

GraphLIME^86^ and LIMEtree^87^ are modifications to LIME as applicable to graph neural networks and regression trees, respectively. LIME has been used in chemistry previously: Whitmore, George, and Hudson^88^ used LIME to explain octane number predictions of molecules from a random forest classifier. Mehdi and Tiwary^89^ used LIME to explain thermodynamic contributions of features. The authors define an “interpretation free energy” which can be achieved by minimizing the surrogate model’s uncertainty and maximizing simplicity. Gandhi and White^10^ used an approach similar to GraphLIME but used chemistry specific fragmentation and descriptors to explain molecular property prediction. Some examples are highlighted in the Applications section.

### Counterfactual Explanations

Counterfactual explanations can be found in many fields such as statistics, mathematics, and philosophy.^90−93^ According to Woodward and Hitchcock,^91^ a counterfactual is an example with minimum deviation from the initial instance but with a contrasting outcome. They can be used to answer the question, “which smallest change could alter the outcome of an instance of interest?” While the difference between the two instances is based on the existence of similar worlds in philosophy,^94^ a distance metric based on molecular similarity is employed in XAI for chemistry. For example, in the work by Wellawatte, Seshadri, and White,^9^ the distance between two molecules is defined as the Tanimoto distance^95^ between ECFP4 fingerprints.^96^ Additionally, Mohapatra, An, and Gómez-Bombarelli^97^ introduced a chemistry-informed graph representation for computing macromolecular similarity. Contrastive explanations are peripheral to counterfactual explanations. Unlike the counterfactual approach, the contrastive approach employs a dual optimization method, which works by generating a similar and a dissimilar (counterfactuals) example. Contrastive explanations can interpret the model by identifying the contribution of the presence and absence of subsets of features toward a certain prediction.^36,98^

A counterfactual *x*′ of an instance *x* is one with a dissimilar prediction *f̂*(*x*) in classification tasks. As shown in eq 5, counterfactual generation can be thought of as a constrained optimization problem which minimizes the vector distance *d*(*x*, *x*′) between the features.^9,99^
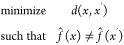
5

For regression tasks, eq 6 adapted from eq 5 can be used. Here, a counterfactual is one with a defined increase or decrease in the prediction.
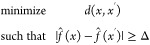
6

Counterfactual explanations have become a useful tool for XAI in chemistry, as they provide an intuitive understanding of predictions and are able to uncover spurious relationships in training data.^100^ Counterfactuals create local (instance-level), actionable explanations. Actionability of an explanation suggests which features can be altered to change the outcome, for example, changing a hydrophobic functional group in a molecule to a hydrophilic group to increase solubility.

Counterfactual generation is a demanding task as it requires gradient optimization over discrete features that represents a molecule. References (101 and 102) introduce two techniques which allow continuous gradient-based optimization. Although these methodologies circumvent the issue of discrete molecular optimization, counterfactual explanation-based model interpretation still remains unexplored compared to other posthoc methods.

CF-GNNExplainer^103^ is a counterfactual explanation generating method based on GNNExplainer^68^ for graph data. This method generates counterfactuals by perturbing the input data (removing edges in the graph) and keeping account of perturbations, which lead to changes in the output. However, this method is only applicable to graph-based models and can generate infeasible molecular structures. Another related work by Numeroso and Bacciu^104^ focuses on generating counterfactual explanations for deep graph networks. Their method, MEG (Molecular counterfactual Explanation Generator), uses a reinforcement learning-based generator to create molecular counterfactuals (molecular graphs). While this method is able to generate counterfactuals through a multiobjective reinforcement learner, this is not a universal approach and requires training the generator for each task.

Work by Wellawatte, Seshadri, and White^9^ presents a model agnostic counterfactual generator named MMACE (Molecular Model Agnostic Counterfactual Explanations), which does not require training or computing gradients. This method first populates a local chemical space through random string mutations of SELFIES^105^ molecular representations using the STONED algorithm.^106^ Next, the labels (predictions) of the molecules in the local space are generated using the model that needs to be explained. Finally, the counterfactuals are identified and sorted by their similarities: Tanimoto distance^95^ between ECFP4 fingerprints.^96^ Unlike the CF-GNNExplainer^103^ and MEG^104^ methods, the MMACE algorithm ensures that generated molecules are valid, owing to the surjective property of SELFIES. Additionally, the MMACE method can be applied to both regression and classification models. However, like most XAI methods for molecular prediction, MMACE does not account for the chemical stability of predicted counterfactuals. To circumvent this drawback, authors propose another approach,^9^ which identifies counterfactuals through a similarity search on the PubChem database.^107^

#### Similarity to Adjacent Fields

Tangential examples to counterfactual explanations are adversarial training and matched molecular pairs. Adversarial perturbations are used during training to deceive the model to expose the vulnerabilities of a model,^108,109^ whereas counterfactuals are applied posthoc. Therefore, the main difference between adversarial and counterfactual examples are in the application, although both are derived from the same optimization problem.^99^ Adversarial Training on EXplanations (ATEX) is another method, which improves model robustness via exposure to adversarial examples.^110^ While there are conceptual disparities, we note that the counterfactual and adversarial explanations are equivalent mathematical objects.

Matched molecular pairs (MMPs) are pairs of molecules that differ structurally at only one site by a known transformation.^111,112^ MMPs are widely used in drug discovery and medicinal chemistry as these facilitate fast and easy understanding of structure–activity relationships.^113−115^ Counterfactuals and MMP examples intersect if the structural change is associated with a significant change in the properties. If the associated changes in the properties are nonsignificant, the two molecules are known as bioisosteres.^116,117^ The connection between MMPs and adversarial training examples has been explored in ref (118). MMPs, which belong to the counterfactual category, are commonly used in outlier and activity cliff detection.^112^ This approach is analogous to counterfactual explanations, as the common objective is to uncover learned knowledge pertaining to structure–property relationships.^69^

## Applications

Model interpretation is certainly not new and a common step in ML in chemistry, but XAI for DL models is becoming more important.^60,65−68,72,87,103,104^ Here, we illustrate some practical examples drawn from our published work on how model-agnostic XAI can be utilized to interpret black-box models and connect the explanations to structure–property relationships. The methods are “Molecular Model Agnostic Counterfactual Explanations” (MMACE)^9^ and “Explaining molecular properties with natural language”.^10^ Then, we demonstrate how counterfactuals and descriptor explanations can propose structure–property relationships in the domain of molecular scent.^31^

### Blood–Brain Barrier Permeation Prediction

The passive diffusion of drugs from the bloodstream to the brain is a critical aspect in drug development and discovery.^119^ Small molecule blood–brain barrier (BBB) permeation is a classification problem routinely assessed with DL models.^120,121^ To explain why DL models work, we trained two models: a random forest (RF) model^122^ and a Gated Recurrent Unit Recurrent Neural Network (GRU-RNN). Then, we explained the RF model with generated counterfactual explanations using the MMACE^9^ and the GRU-RNN with descriptor explanations.^10^ Both the models were trained on the data set developed by Martins, Teixeira, Pinheiro, and Falcao.^123^ The RF model was implemented in Scikit-learn^124^ using Mordred molecular descriptors^125^ as the input features. The GRU-RNN model was implemented in Keras.^126^ See refs (9 and 10) for further details.

**Figure 1:**
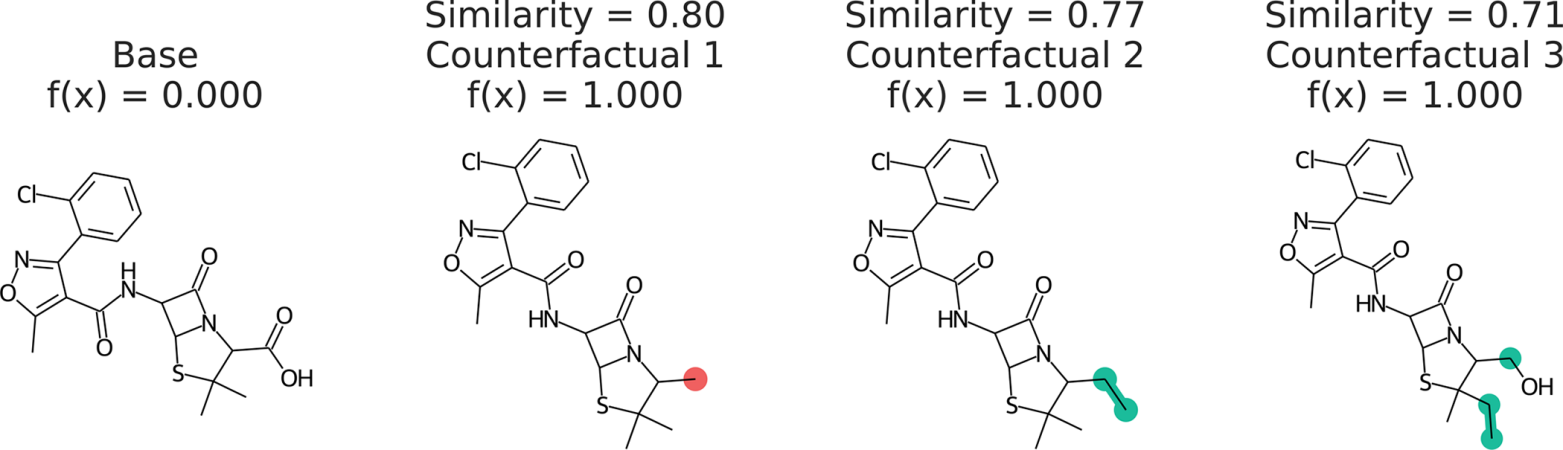
Counterfactuals of a molecule which cannot permeate the blood–brain barrier. Similarity is the Tanimoto similarity of ECFP4 fingerprints.^130^ Red indicates deletions and green indicates substitutions and addition of atoms. Republished from ref (9) with permission from the Royal Society of Chemistry. Copyright 2022.

According to the counterfactuals of the instance molecule in Figure 1, we observe that the modifications to the carboxylic acid group enable the negative example molecule to permeate the BBB. Experimental findings show that the BBB permeation of molecules are governed by hydrophobic interactions and surface area.^119^ The carboxylic group is a hydrophilic functional group which hinders hydrophobic interactions, and the addition of atoms enhances the surface area. This proves the advantage of using counterfactual explanations, as they suggest actionable modification to the molecule to make it cross the BBB.

**Figure 2:**
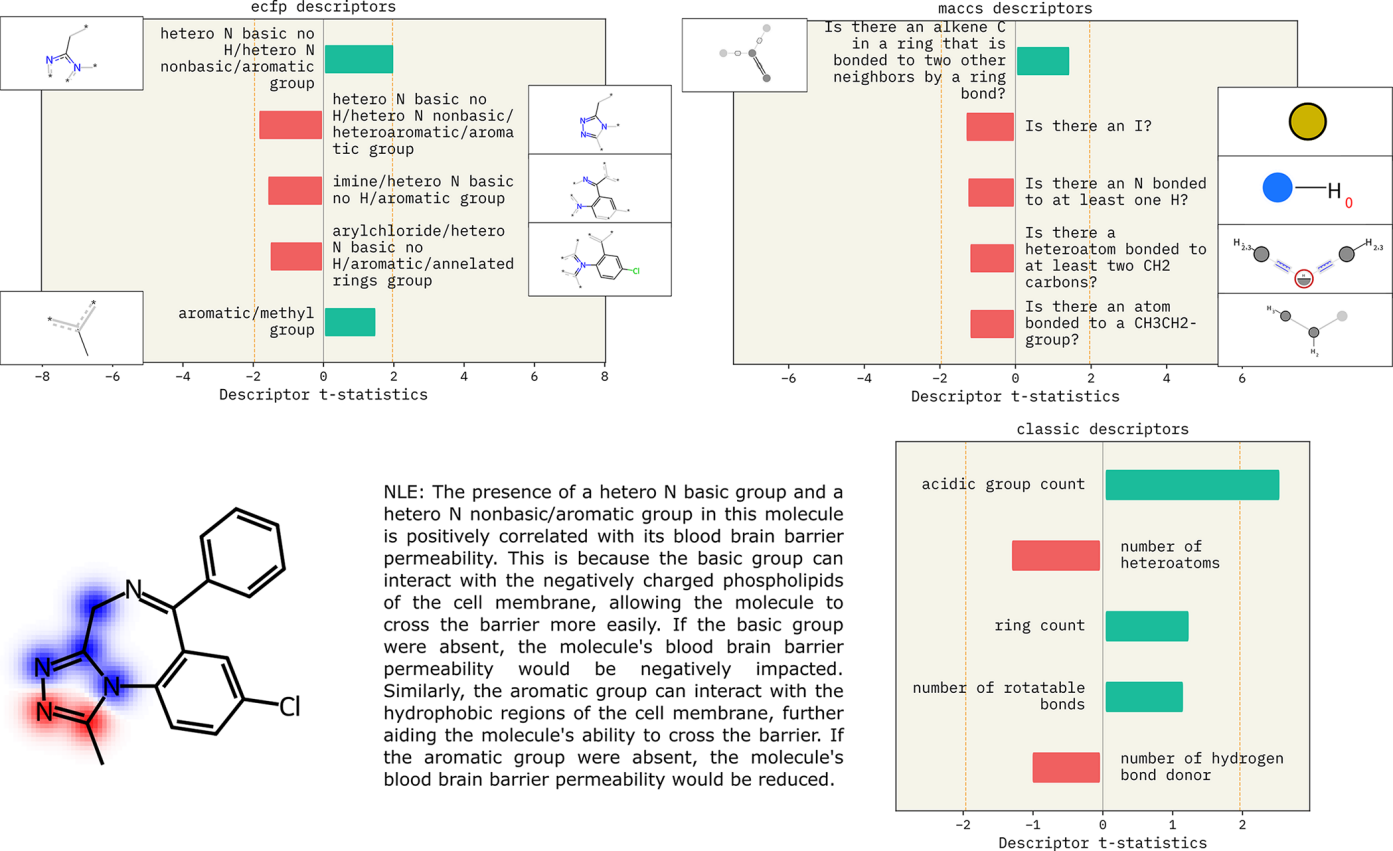
Descriptor explanations along with natural language explanation obtained for BBB permeability of the Alprozolam molecule. The green and red bars show descriptors that influence predictions positively and negatively, respectively. Dotted yellow lines show the significance threshold (α = 0.05) for the t-statistic. Molecular descriptors show molecule-level properties that are important for the prediction. ECFP and MACCS descriptors indicate which substructures influence model predictions. MACCS explanations lead to text explanations as shown. Republished from ref (10) with permission from authors. Copyright 2022. SMARTS annotations for MACCS descriptors were created using SMARTSviewer (https://smarts.plus/smartsview; Copyright: ZBH, Center for Bioinformatics Hamburg).^131^

In Figure 2, we show descriptor explanations generated for Alprozolam, a molecule that permeates the BBB, using the method described in ref (10). We see that predicted permeability is positively correlated with the aromaticity of the molecule, while negatively correlated with the number of hydrogen bond donors and heteroatom count. A similar structure–property relationship for BBB permeability is proposed in more mechanistic studies.^127−129^ The substructure attributions indicate a reduction in hydrogen bond donors and acceptors. These descriptor explanations are quantitative and interpretable by chemists. Finally, we can use a natural language model to summarize the findings into a written explanation, as shown in the printed text in Figure 2.

### Solubility Prediction

Small molecule solubility prediction is a classic cheminformatics regression challenge and is important for chemical process design, drug design, and crystallization.^132−135^ In our previous works,^9,10^ we implemented and trained an RNN model in Keras to predict solubilities (log molarity) of small molecules.^126^ The AqSolDB curated database^136^ was used to train the RNN model.

**Figure 3:**
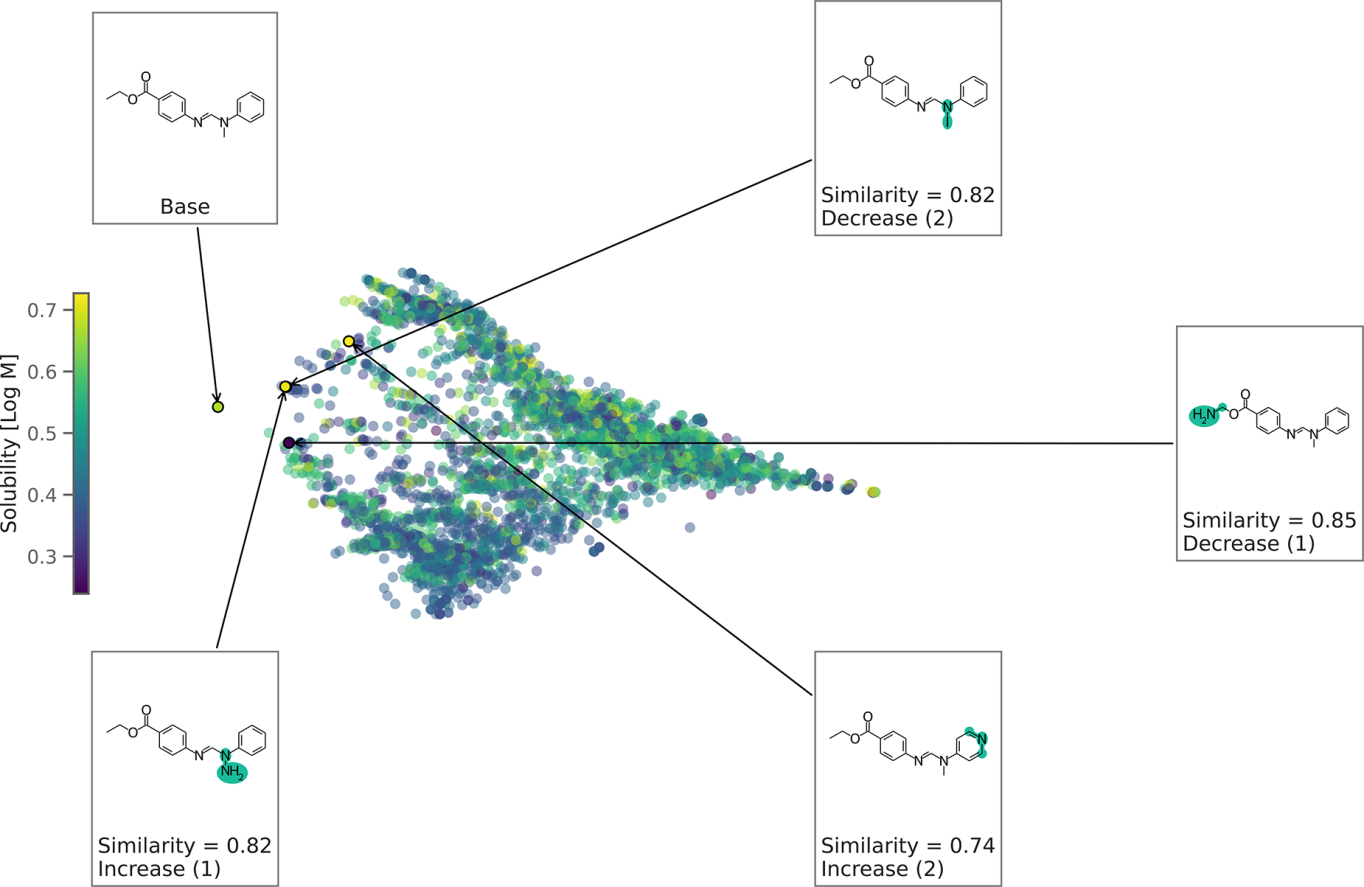
Generated chemical space for solubility prediction using the RNN model. The chemical space is a 2D projection of the pairwise Tanimoto similarities of the local counterfactuals. Each data point is colored by solubility. The top 4 counterfactuals are shown here. Republished from ref (9) with permission from the Royal Society of Chemistry. Copyright 2022.

**Figure 4:**
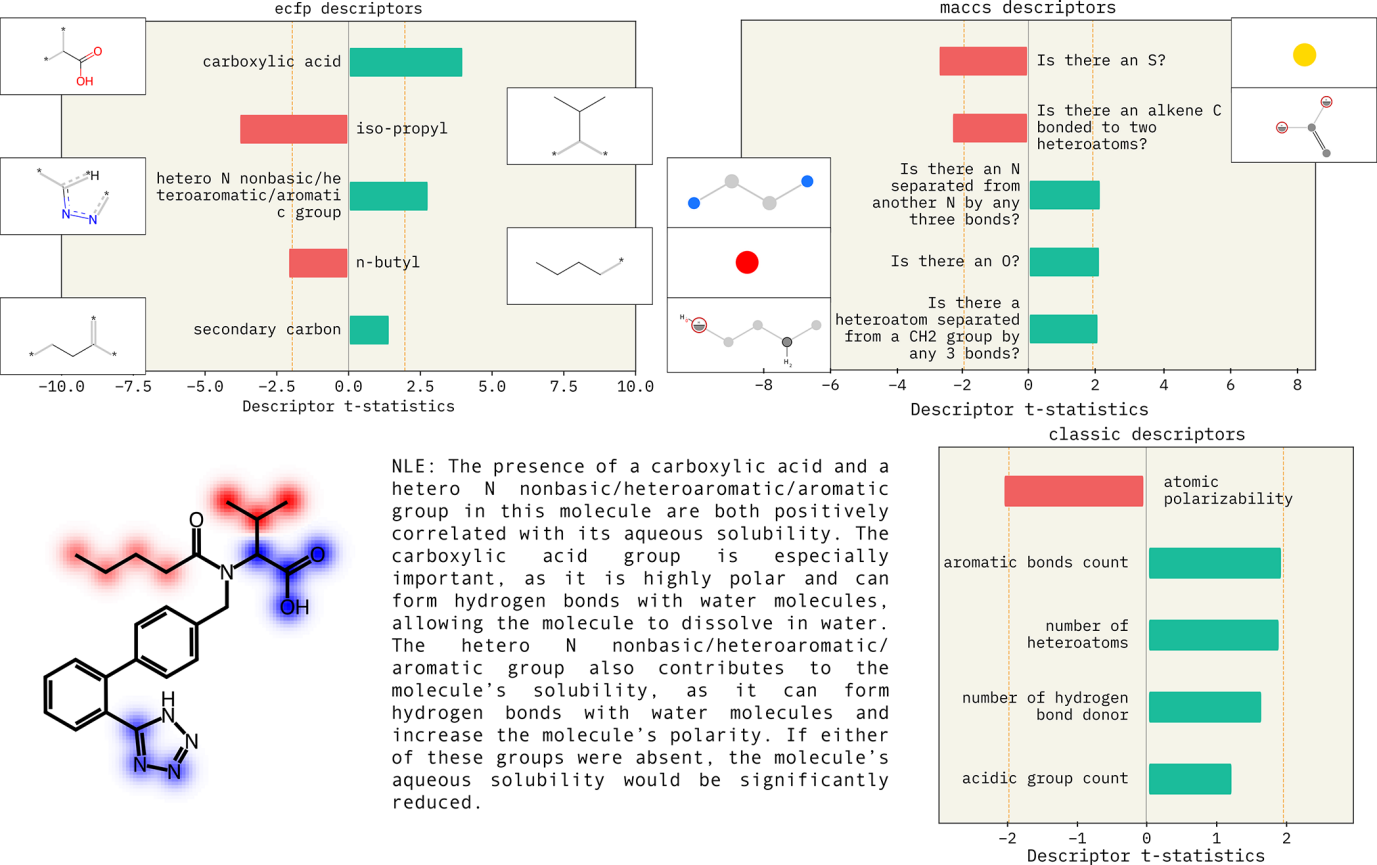
Descriptor explanations for solubility prediction model. The green and red bars show descriptors that influence predictions positively and negatively, respectively. Dotted yellow lines show the significance threshold (α = 0.05) for the t-statistic. The MACCS and ECFP descriptors indicate which substructures influence model predictions. MACCS substructures may either be present in the molecule as is or represent a modification. ECFP fingerprints are substructures in the molecule that affect the prediction. MACCS descriptors are used to obtain text explanations as shown. Republished from ref (10) with permission from authors. Copyright 2022. SMARTS annotations for MACCS descriptors were created using SMARTSviewer (https://smarts.plus/smartsview; Copyright: ZBH, Center for Bioinformatics Hamburg).^131^

In this task, counterfactuals are based on eq 6. Figure 3 illustrates the generated local chemical space and the top four counterfactuals. Based on the counterfactuals, we observe that the modifications to the ester group and other heteroatoms play an important role in solubility. These findings align with known experimental and basic chemical intuition.^133^Figure 4 shows a quantitative assessment of how ECFP^96^ and MACCS^137^ substructures are contributing to a prediction. We observe that the presence of carboxylic acid and heteroatomic groups are positively correlated with solubility. The descriptor attributions are clearly described by the natural language explanation (NLE) in the method introduced by Gandhi and White.^10^ Furthermore, the text gives rationale behind predictions. For example, in Figure 4, we explain why having a carboxylic group is important: “it is highly polar and can form hydrogen bonds with water molecules”. This result shows that descriptor attributions with NLE can be used to make predictions from black-box models human interpretable.

### Generalizing XAI: Interpreting Scent–Structure Relationships

In this example, we show how nonlocal structure–property relationships can be learned with XAI across multiple molecules. Molecular scent prediction is a multilabel classification task because a molecule can be described by more than one scent. For example, the molecule jasmone can be described as having “jasmine”, “woody”, “floral”, and “herbal” scents.^138^ The scent–structure relationship is not very well understood,^139^ although some relationships are known. For example, molecules with an ester functional group are often associated with the “fruity” scent. There are some exceptions though, like *tert*-amyl acetate, which has a “camphoraceous” rather than “fruity” scent.^139,140^

In Seshadri, Gandhi, Wellawatte, and White,^31^ we trained a GNN model to predict the scent of molecules and utilized counterfactuals^9^ and descriptor explanations^10^ to quantify scent–structure relationships. The MMACE method was modified to account for the multilabel aspect of scent prediction. This modification defines molecules that differed from the instance molecule by only the selected scent as counterfactuals. For instance, counterfactuals of the jasmone molecule would be false for the “jasmine” scent but would still be positive for “woody”, “floral”, and “herbal” scents.

**Figure 5:**
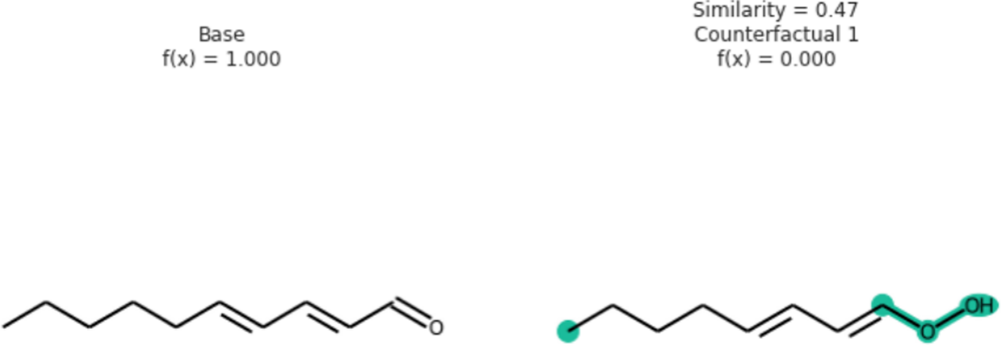
Counterfactual for the 2,4 decadienal molecule. The counterfactual indicates structural changes to this molecule that would result in the model predicting the molecule to not contain the “fatty” scent. The Tanimoto^95^ similarity between the counterfactual and 2,4 decadienal is 0.47. Republished with permission from authors.^31^

The molecule 2,4-decadienal, which is known to have a “fatty” scent, is analyzed in Figure 5.^141,142^ The resulting counterfactual, which has a shorter carbon chain and no carbonyl groups, highlights the influence of these structural features on the “fatty” scent of 2,4-decadienal.

To generalize to other molecules, we applied the descriptor attribution method by Gandhi and White^10^ to obtain global explanations for the scents. The global explanation for the “fatty” scent was generated by gathering chemical spaces around many “fatty” scented molecules. The resulting natural language explanation is “The molecular property ‘fatty scent’ can be explained by the presence of a heptanyl fragment, two CH2 groups separated by four bonds, and a C=O double bond, as well as the lack of more than one or two O atoms.”^31^ The importance of a heptanyl fragment aligns with that reported in the literature, as “fatty” molecules often have a long carbon chain.^143^ Furthermore, the importance of a C=O double bond is supported by the findings reported in ref (144), where in addition to a “larger carbon-chain skeleton”, they found that “fatty” molecules also had “aldehyde or acid functions”.^144^ For the “pineapple” scent, the following natural language explanation was obtained: “The molecular property “pineapple scent” can be explained by the presence of ester, ethyl/ether O group, alkene/ether O group, and C=O double bond, as well as the absence of an Aromatic atom.”^31^ Esters, such as ethyl 2-methylbutyrate, are present in many pineapple volatile compounds.^145,146^ The combination of a C=O double bond with an ether could also correspond to an ester group. Additionally, aldehydes and ketones, which contain C=O double bonds, are also common in pineapple volatile compounds.^145,147^

## Discussion

We have shown two posthoc XAI applications based on molecular counterfactual explanations^9^ and descriptor explanations.^10^ These methods can be used to explain black-box models whose input is a molecule. These two methods can be applied for both classification and regression tasks. Note that the “correctness” of the explanations strongly depends on the accuracy of the black-box model.

A molecular counterfactual is one with a minimal distance from a base molecule but with contrasting chemical properties. In the above examples, we used Tanimoto similarity^95^ of ECFP4 fingerprints^96^ as distance, although this should be explored in the future. Counterfactual explanations are useful because they are represented as chemical structures (familiar to domain experts), sparse, and actionable. A few other popular examples of counterfactual on *graph* methods are GNNExplainer, MEG, and CF-GNNExplainer.^68,103,104^

The descriptor explanation method developed by Gandhi and White^10^ fits a self-explaining surrogate model to explain the black-box model. This is similar to the GraphLIME^86^ method, although we have the flexibility to use explanation features other than subgraphs. Futhermore, we show that natural language combined with chemical descriptor attributions can create explanations useful for chemists, thus enhancing the accessibility of DL in chemistry. Lastly, we examined if XAI can be used beyond interpretation. Work by Seshadri, Gandhi, Wellawatte, and White^31^ used MMACE and descriptor explanations to analyze the structure–property relationships of scent. They recovered known structure–property relationships for molecular scent purely from explanations, demonstrating the usefulness of a two-step process: fit an accurate model and then explain it.

Choosing among the plethora of XAI methods described here is still an open question. It remains to be seen if there will ever be a consensus benchmark, since this field sits at the intersection of human–machine interaction, machine learning, and philosophy (i.e., what constitutes an explanation?). Our current advice is to consider first the audience, domain experts or ML experts or nonexperts, and what the explanations should accomplish. Are they meant to inform data selection or model building, how a prediction is used, or how the features can be changed to affect the outcome. The second consideration is what access you have to the underlying model. The ability to have model derivatives or propagate gradients to the input to models also informs the XAI method.

## Conclusion and Outlook

We should seek to explain molecular property prediction models because users are more likely to trust explained predictions and explanations can help assess if the model is learning the correct underlying chemical principles. We also showed that black-box modeling first, followed by XAI, is a path to structure–property relationships without needing to trade between accuracy and interpretability. However, XAI in chemistry has some major open questions that are also related to the black-box nature of the deep learning models. Some are highlighted below:

- *Explanation representation*: How is an explanation presented: text, a molecule, attributions, a concept, etc?
- *Molecular distance*: In XAI approaches such as counterfactual generation, the “distance” between two molecules is minimized. Molecular distance is subjective. Possibilities are distance based on molecular properties, synthesis routes, and direct structure comparisons.
- *Regulations*: As black-box models move from research to industry, healthcare, and environmental settings, we expect XAI to become more important to explain decisions to chemists or nonexperts and possibly be legally required. Explanations may need to be tuned for doctors instead of chemists or to satisfy a legal requirement.
- *Chemical space*: Chemical space is the set of molecules that are realizable; “realizable” can be defined from purchasable to synthesizable to satisfied valencies. What is most useful? Can an explanation consider nearby impossible molecules? How can we generate local chemical spaces centered around a specific molecule for finding counterfactuals or other instance explanations? Similarly, can “activity cliffs” be connected to explanations and the local chemical space.^148^
- *Evaluating XAI*: There is a lack of a systematic framework (quantitative or qualitative) to evaluate correctness and applicability of an explanation. Can there be a universal framework or should explanations be chosen and evaluated based on the audience and domain? There have been few attempts at bridging this gap. For example, work in ref (58) presents a benchmark on comparing feature attribution XAI methods via Crippen’s logP scores.
